# Parental Attitudes and Practices regarding Atopic Dermatitis: A Cross-Sectional Study among a Thai Population

**DOI:** 10.3390/children11070870

**Published:** 2024-07-18

**Authors:** Phurithat Nummak, Leelawadee Techasatian, Rattapon Uppala, Phanthila Sitthikarnkha, Suchaorn Saengnipanthkul, Prapassara Sirikarn

**Affiliations:** 1 Department of Pediatrics, Faculty of Medicine, Khon Kaen University, Khon Kaen 40002, Thailand; chanitkarnla@kkumail.com (P.N.); rattapon@kku.ac.th (R.U.); puntsi@kku.ac.th (P.S.); suchsa@kku.ac.th (S.S.); 2 Department of Epidemiology and Biostatistics, Faculty of Public Health, Khon Kaen University, Khon Kaen 40002, Thailand; prapsiri@kku.ac.th

**Keywords:** atopic dermatitis, children, parents, attitude, knowledge, practice

## Abstract

Background: Atopic dermatitis (AD) is a chronic inflammatory skin disorder common in children. Successful pediatric AD therapy requires parental assistance. Thus, evaluating parental knowledge, attitudes, and behaviors regarding childhood AD may lead to more educational recommendations to help children control AD in the future. This study examined parents’ knowledge, attitudes, and conduct concerning AD in families with and without children with AD. Method: The Pediatric Department, Faculty of Medicine, Khon Kaen University, Thailand, conducted a cross-sectional study from June to December 2023. Parents of children who visited the dermatology clinic with or without AD were asked to complete a Google form questionnaire. Results: A total of 372 parents answered a questionnaire about AD pathophysiology, knowledge, attitudes, and practices. The participants were 293 (78.8%) female participants and 79 (21.2%) male participants. The average age was 29.79 (SD 4.91). Most parents (319, 85.8%) did not work in the medical field, and more than half (228 instances, 61.29%) had children diagnosed with AD. Conclusions: Parents of children with AD understood AD causes and triggers better than parents of children without AD. But, “exposure to furry toys” that may contain dust and allergies and “infection” that may cause AD flare-ups were the most common triggers, regardless of the group. Appropriate information should be supplied because both the parents of children with AD and those of children without AD reported immediate food avoidance without confirmatory testing, which might lead to malnutrition. Clinicians and families handling patients with AD require further education.

## 1. Background

Atopic dermatitis (AD) is a chronic inflammatory skin condition characterized by itchy, inflamed skin that flares up, relapses, and persists over time [1]. It is a common skin condition that commonly occurs in childhood and may last into adulthood. Like many chronic diseases, affected patients may face a number of disadvantages and challenges in their everyday lives [2]. Atopic dermatitis (AD) in children is associated with a significant disease burden, impacting not only the child but also their family and caregivers [3]. The burden of AD in pediatric patients is characterized by a multidimensional impact on various aspects of their lives, including itch, pain, sleep disturbance, impaired health-related quality of life (HRQoL), and the presence of atopic comorbidities [4]. Constant discomfort and painful skin fissures from skin inflammation, sleep issues from overnight itching, impact on daily activities, psychological effects, social and educational isolation, financial challenges, and stigma are some of the downsides of AD [5]. Moreover, patients with AD usually have some complications, including skin infections, scarring, and other skin issues which may require additional medical attention and treatment [6]. Due to several complications and the chronicity of the disease, the parents of children with atopic dermatitis tend to seek several medical consultations, leading to high costs [7]. Some parents may experience a lack of disease understanding and even adopt malpractices when treating this chronic skin condition. This may even make the disease worse and uncontrolled.

Understanding parental knowledge, attitudes, and behaviors regarding childhood AD may lead to more educational recommendations that will aid in the successful control of AD in children in the future. However, few studies have investigated these characteristics in a Thai setting. As a result, the primary goal of the current study was to investigate parental knowledge, attitudes, and practices related to atopic dermatitis in Thailand. This study’s findings may assist in illuminating Thailand’s unique cultural and socioeconomic context, improving disease treatment and quality of life for children with AD and their families.

## 2. Objectives

The aim of this study was to investigate parents’ knowledge, attitudes, and behaviors regarding AD among parents of children with and without AD. Parents’ attitudes and practices are essential for enhancing patient care, improving the quality of life of those affected by AD, promoting prevention and education, supporting the psychological well-being of parents, and addressing public health challenges related to this condition.

## 3. Design and Method

A cross-sectional study was conducted at the Pediatric Department, Faculty of Medicine, Khon Kaen University, Thailand, between June 2023 and December 2023. Parents of children who consecutively visited the dermatologic clinic with or without AD were invited to complete a questionnaire via a Google form. The questionnaire comprised four sections: demographic data, general knowledge, practices, and attitudes toward AD. This study was approved by the institutional review board of Khon Kaen University’s Faculty of Medicine (IRB No. HE661258). This research was a part of Cho Kalaphruek Excellent Research Projects for Medical Students and was supported by Research and Graduate Studies, Khon Kaen University, Thailand.

## 4. Statistical Analysis

The characteristics of the participants were presented as a frequency and a percentage for categorical data and as a mean with standard deviation (SD) and a median with an interquartile range (IQR) for continuous data. The characteristics of the participants were compared between groups with an independent t-test for continuous data and a chi-squared test or Fisher’s exact test for categorical data. The factors associated with the children diagnosed with AD were analyzed using both simple and multiple logistic regression with a backward, stepwise elimination method and reported as the adjusted odds ratio (OR) with a 95% confidence interval (CI). We compared the attitudes towards AD between groups using Pearson’s chi-squared test. All statistical analyses were performed using STATA 15.0.

## 5. Results

A total of 372 parents completed a questionnaire addressing their knowledge regarding the pathogenesis of atopic dermatitis (AD), basic skin-care practices, and the perceived role of infection and food allergies in AD. There were 293 (78.8%) female and 79 (21.2%) male participants. The mean age was 29.79 years (SD 4.91). The majority of the parents (319, 85.8%) did not work in a medical field, and more than half of the participants (228 cases, 61.29%) had children with a diagnosis of AD. The demographic data of the parents are shown in Table 1.

There was a question asking the parents to select more than one choice from a list of possible causes of AD. The parents picked “the problem with the immune system” (219, 42.7%), “food allergy” (213, 42.7%), “genetic” (130, 34.9%), and “dry skin” (128, 34.4%). When compared with the parents of children without AD, the parents of children with AD selected causes such as “food allergy” and “dry skin” 7.4 times (adjust OR 7.40, 95% CI 4.38–12.50, *p* < 0.001) and 5.59 times more (adjust OR 5.59, 95% CI 3.27–9.56, *p* < 0.001), as shown in Table 2.

The questionnaire indicated several trigger factors for AD, including infection, bathing in hot water, sweat and a hot environment, cold and dry environment, furry pets, furry toys, scratching, and stress. Table 2 provides the responses selected by the participating parents. The parents of children with AD selected considerably more trigger items that they perceived to be worsening AD than the parents of children without AD, with the exception of “infection” and “furry toys”, which revealed no significant differences between the two parent groups.

The lists of common practices for patients with AD included enhancing breast feeding in AD prevention, applying moisturizer, and immediate food avoidance in a child, without a proven test. In these “common practice” questions, the parents of children with AD and the parents of children without AD revealed no difference in their routine practices, as shown in Table 3.

**Figure 1:**
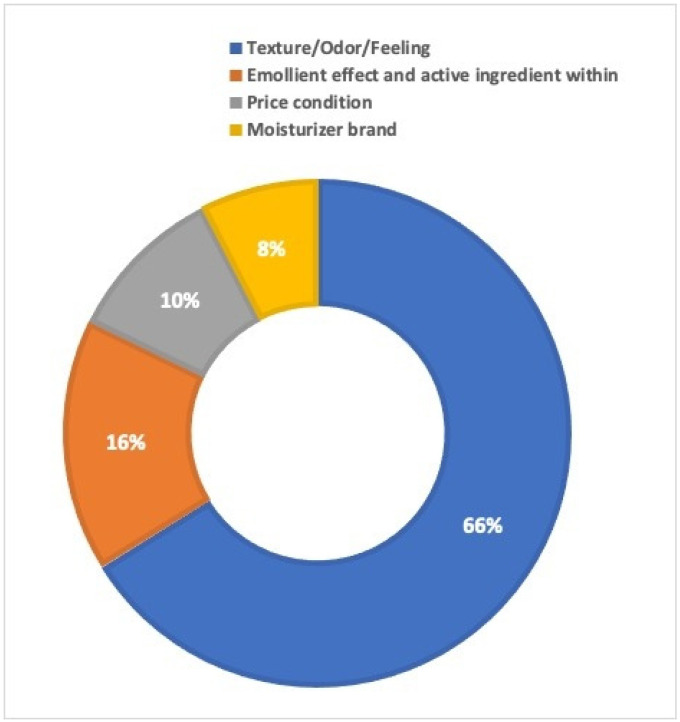
The decision-making aspects involved in moisturizer selection among the study population.

Applying moisturizer was a common practice chosen among the various ones listed in questionnaire. This was more common among the parents of children with AD (178, 78.1%), as shown in Table 3. The majority choose the moisturizer based on their preferences for texture/odor and sensation after application (246, 66.1%), followed by emollient action and active ingredients (60, 16.1%), price (38, 10.2%), and brand (28, 7.5%). Figure 1 depicts the decision-making aspects involved in moisturizer selection among the study population.

**Figure 2:**
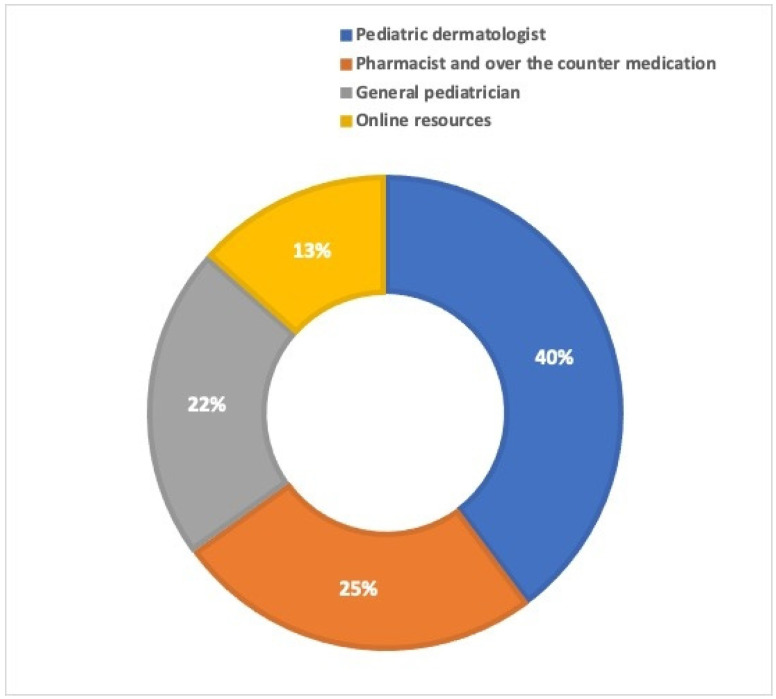
The sources that participating parents sought guidance from.

The majority of the participating parents (148, 39.8%) sought advice from a specialist, particularly a pediatric dermatologist, regarding their child’s skin health. This was followed by consulting a pharmacist and using over-the-counter medicine (94, 25.3%), seeking guidance from a general pediatrician (80, 21.5%), and utilizing internet resources (50, 13.4%), as shown in Figure 2.

## 6. Discussion

The efficacy of therapy for pediatric atopic dermatitis is greatly impacted by the parents’ attitude and understanding of the problem [8]. This may be contradictory to the preferences of teenagers and adults, who may have the ability to choose how they treat the disease themselves [9]. Therefore, understanding the attitude and knowledge of AD among parents may lead to a better approach to pediatric AD treatment. The present study revealed the very first results regarding Thai parents’ understanding and knowledge of AD in families of children both with and without AD. The results revealed that the parents of children with AD had a better knowledge about the causes and triggers of AD in many areas. This finding is similar to previous works in the literature that found that parents with personal AD experience and those with children who had previously had AD were more knowledgeable and had stronger positive attitudes about AD [8].

This simply means that the parents of children with AD are more aware of the disease and seek out information about this chronic skin condition than the parents of children without AD. However, the current study revealed that, while the parents of children with AD were more knowledgeable about AD than those of without the disease, there were some points that the parents of children with AD may have overlooked, as there was no difference in some typical trigger variables which parents should be aware of, such as an awareness of an “infection” which could trigger AD.

Infection is a frequent cause of AD flare-ups. Recurrent bacterial and viral skin infections are prevalent in AD, and, if left untreated, they can lead to significant complications [10]. Other common infections in children, such as respiratory and gastrointestinal infections, can potentially cause an AD flare-up. Patients with atopic dermatitis are more likely to suffer respiratory infections than those with non-atopic diseases. A previous study has also indicated that patients with atopic diseases, including AD, have significantly more upper and lower respiratory tract infections than individuals with non-atopic diseases, owing to a susceptibility to respiratory infections associated with the presence of specific IgE antibodies [11]. As part of secondary AD prevention, parents should be taught how to avoid infections that might lead to disease flare-ups in children.

Aeroallergens, including indoor aeroallergens such as house dust mites (HDMs), pet dander, fur, and cockroaches, can trigger AD [12]. The present study discovered that knowledge and practice of this key aspect of AD trigger avoidance is also lacking among parents of children with AD. Most parents are aware that children with furry pets are at greater risk of developing AD, but they are less aware that furry toys can also provoke an AD flare-up. In terms of secondary AD prevention, all potential trigger factors should be highlighted and included as information for all parents of children with AD in the future.

Food allergies have been identified as a factor contributing to the development of AD. Food allergy-related AD is typically observed in young children and is characterized by moderate-to-severe symptoms. Prior to implementing food avoidance, it is necessary to perform a confirmatory test for those with food allergies who are also diagnosed with AD [6]. Following treatment suggestions can assist parents in reducing unnecessary food avoidance. However, in real-life situations, many people exhibit aggravated attentiveness and concern towards this issue. When patients have a flare-up of AD, their initial assumption will often be that a food allergy was causative. Even though there is clear standard guidance against avoiding food before confirmatory tests, the majority of patients opt to do so. The present study discovered that both the parents of children with AD and those of children without AD had the same preference to avoid particular foods in the absence of validated diagnostic tests. This behavior may lead to unnecessary food avoidance and, eventually, malnutrition. As a result, all patients and caregivers should be promptly advised of this important information [13] in order to avoid this potentially harmful practice in the future.

The initial step in AD treatment is to apply moisturizer. This approach has been proven to improve the skin barrier, which is one of the key pathologies in AD. There are several moisturizers on the market, some containing extra active ingredients which claim to be more beneficial for patients with AD [14], commonly referred to as “therapeutic moisturizers” or “moisturizer plus”.

A recent publication revealed that different emollient creams have different effects on our skin, and only certain types have the ability to improve the skin’s barrier and protect against the irritants that trigger eczema [14]. Another study also revealed corticosteroid-sparing in the moisturizer “plus” group compared to the control group in terms of quantity [15]. These findings may support the preference for moisturizers with additional active components that are appropriate for patients with AD over basic moisturizers. However, in addition to these active components, the price of these products is also increased.

The present study found that the most common reason among the study group for picking a moisturizer was the “texture/odor of the product” rather than the understanding of the specific ingredients included. This may represent an awareness of various moisturizer types, which may indicate a knowledge deficit to be addressed in future education efforts regarding patients with AD. According to the current study, the most common answer for how to pick a moisturizer in AD was the “texture/odor of the product”; hence, optimizing moisturizer treatment for skin barrier repair in AD should still be based on patient desire. Other essential factors to consider while selecting a moisturizer were the severity of dryness of the patient’s skin and the environment in which they resided. In hot-temperature countries, particularly during the summer, using too much moisturizer can cause skin problems like miliaria, especially in young children [16]. As a result, we strongly believe that choosing a moisturizer should always be based on this important information, in addition to the benefits of “Moisturize Plus” products [17].

There are several research limitations. For example, due to the layout of the questionnaire, some of the answers provided by the parents may have needed clarification, such as the reason why they chose a specific response; hence, an in-depth interview study may give more depth and clarification regarding these topics. Furthermore, the severity of the disease in patients with AD was not considered in the current study; nevertheless, knowing the various levels of severity of individual patients with AD may explain more differences in parental attitudes and practices. 

## 7. Conclusions

The parents of children with AD had a better understanding of the causes and triggers of AD than the parents of children without AD. However, “exposure to furry toys” that may include dust and allergens as well as “infection” that may produce AD flare-ups were two of the most prevalent triggers, with no difference across groups. Immediate food avoidance without confirmatory testing was reported in the parents of both children with and without AD, which might lead to unnecessary food avoidance and malnutrition; hence, adequate information should be included. Future educational programs and better ways to help people engage in healthy behaviors to prevent the onset or worsening of chronic disease should be available for the clinicians and families who deal with patients with AD.

## Figures and Tables

**Table 1:** Participating parents’ characteristics.

Characteristics	Total (n = 372)		No Children with a Diagnosis of AD (n = 144)		Children with a Diagnosis of AD (n = 228)		*p*-Value
	n	(%)	n	%	n	%	
Sex							
Female	293	(78.8%)	121	(84.0%)	172	(75.4%)	0.048
Male	79	(21.2%)	23	(16.0%)	56	(24.6%)	
Age (years)							
Mean (SD)	29.79	(4.91)	27.27	(2.72)	31.38	(5.31)	<0.001
Occupation							
Healthcare worker	53	(14.2%)	1	(0.7%)	52	(22.8%)	<0.001
Not a healthcare worker	319	(85.8%)	143	(99.3%)	176	(77.2%)	
Highest level of education							
High school/ high school diploma	38	(10.2%)	25	(17.3%)	13	(5.7%)	<0.001
Associate’s degree	137	(36.8%)	58	(40.3%)	79	(34.7%)	
Bachelor’s degree	188	(50.6%)	60	(41.7%)	128	(56.1%)	
Professional degree	9	(2.4%)	1	(0.7%)	8	(3.5%)	
Marital status							
Not married	88	(23.7%)	60	(41.7%)	28	(12.3%)	<0.001
Currently married	227	(61.0%)	84	(58.3%)	143	(62.7%)	
Divorced	37	(9.9%)	0	(0.0%)	37	(16.2%)	
No answer	20	(5.4%)	0	(0.0%)	20	(8.8%)	
Household income/ month (THB)							
<15,000	124	(33.3%)	78	(54.2%)	46	(20.2%)	<0.001
15,000–30,000	198	(53.2%)	48	(33.3%)	150	(65.8%)	
30,000–50,000	41	(11.0%)	17	(11.8%)	24	(10.5%)	
50,000–100,000	0	(0.0%)	0	(0.0%)	0	(0.0%)	
>100,000	9	(2.4%)	1	(0.7%)	8	(3.5%)	
Number of children							
1	268	(72.0%)	118	(81.9%)	150	(65.8%)	<0.001
2	102	(27.4%)	24	(16.7%)	78	(34.2%)	
3	2	(0.6%)	2	(1.4%)	0	(0.0%)	
Mean (SD)	1.28	(0.46)	1.19	(0.43)	1.34	(0.48)	

**Table 2:** Attitude and perception of the causes and triggers of atopic dermatitis among participating parents.

Response	Total (n = 372)		No Children with a Diagnosis of AD (n = 144)		Children with a Diagnosis of AD (n = 228)		Adjusted OR (95% CI)	*p*-Value
	n	(%)	n	(%)	n	(%)		
Causes of AD								
Dry skin								<0.001
No	244	(65.6%)	118	(81.9%)	126	(55.3%)	1	
Yes	128	(34.4%)	26	(18.1%)	102	(44.7)	5.59 (3.27 to 9.56)	
Food allergy								<0.001
No	213	(57.3%)	114	(79.2%)	99	(43.4%)	1	
Yes	159	(42.7%)	30	(20.8%)	129	(56.6%)	7.40 (4.38 to 12.50)	
Genetic								0.882
No	242	(65.1%)	100	(69.4%)	142	(62.3%)	1	
Yes	130	(34.9%)	44	(30.6%)	86	(37.7%)	1.04 (0.63 to 1.70)	
Problem with immune response								0.022
No	153	(41.1%)	71	(49.3%)	82	(36.0%)	1	
Yes	219	(58.9%)	73	(50.7%)	146	(64.0%)	1.65 (1.07 to 2.54)	
Triggered factors								
Infection								0.692
No	123	(33.1%)	48	(33.3%)	75	(32.9%)	1	
Yes	249	(66.9%)	96	(66.7%)	153	(67.1%)	1.10 (0.70 to 1.72)	
Hot bathing								<0.001
No	131	(35.2%)	77	(53.5%)	54	(23.7%)	1	
Yes	241	(64.8%)	67	(46.5%)	174	(76.3%)	3.59 (2.17 to 5.93)	
Sweats and hot environment								<0.001
No	153	(41.1%)	90	(62.5%)	63	(27.6%)	1	
Yes	219	(58.9%)	54	(37.5%)	165	(72.4%)	4.82 (2.83 to 8.22)	
Cold and dry environment								<0.001
No	116	(31.2%)	71	(49.3%)	45	(19.7%)	1	
Yes	256	(68.8%)	73	(50.7%)	183	(80.3%)	4.09 (2.51 to 6.68)	
Furry pets								0.003
No	86	(23.1%)	48	(33.3%)	38	(16.7%)	1	
Yes	286	(76.9%)	96	(66.7%)	190	(83.3%)	2.17 (1.29 to 3.64)	
Furry toys								0.523
No	106	(28.5%)	48	(33.3%)	58	(25.4%)	1	
Yes	266	(71.5%)	96	(66.7%)	170	(74.6%)	1.17 (0.72 to 1.92)	
Stress								0.024
No	50	(13.4%)	29	(20.1%)	21	(9.2%)	1	
Yes	322	(86.6%)	115	(79.9%)	207	(90.8%)	2.05 (1.10 to 3.84)	
Scratching								<0.001
No	72	(19.4%)	47	(32.6%)	25	(11.0%)	1	
Yes	300	(80.6%)	97	(67.4%)	203	(89.0%)	4.93 (2.69 to 9.01)	

**Table 3:** Practices in participating parents of children with and without AD.

Response	Total (n = 372)		No Children with a Diagnosis of AD (n = 144)		Children with a Diagnosis of AD (n = 228)		Adjusted OR (95% CI)	*p*-Value
	n	(%)	n	(%)	n	(%)		
Practice								
Breast feeding for AD prevention								0.093
No	139	(37.4%)	65	(45.1%)	74	(32.5%)	1	
Yes	233	(62.6%)	79	(54.9%)	154	(67.5%)	1.47 (0.94 to 2.30)	
Apply moisturizer								0.125
No	98	(26.3%)	48	(33.3%)	50	(21.9%)	1	
Yes	274	(73.7%)	96	(66.7%)	178	(78.1%)	1.48 (0.90 to 2.43)	
Maternal food avoidance during lactation and breastfeeding								0.650
No	152	(40.9%)	61	(42.4%)	91	(39.9%)	1	
Yes	220	(59.1%)	83	(57.6%)	137	(60.1%)	1.10 (0.72 to 1.70)	
Food avoidance in a child immediately when suspected, without a confirmed test								0.135
No	216	(58.1%)	91	(63.2%)	125	(54.8%)	1	
Yes	156	(41.9%)	53	(36.8%)	103	(45.2%)	1.40 (0.90 to 2.16)	
Apply moisturizer								0.125
No	98	(26.3%)	48	(33.3%)	50	(21.9%)	1	
Yes	274	(73.7%)	96	(66.7%)	178	(78.1%)	1.48 (0.90 to 2.43)	

## Data Availability

Data availability is available in the Supplementary Materials.

## Supplementary Materials

The following supporting information can be downloaded at https://www.mdpi.com/article/10.3390/children11070870/s1.
